# Total sugar intake is associated with higher prevalence of depressive symptoms in obese adults

**DOI:** 10.3389/fpubh.2022.1069162

**Published:** 2023-01-13

**Authors:** Ping Li, Fuzai Yin, Yanru Zhao, Yali Liu, Ru Zhang, Jia Wang, Wenqi Lu, Qingsheng Wang, Jing Zhang

**Affiliations:** ^1^Department of Nutrition, First Hospital of Qinhuangdao, Qinhuangdao, China; ^2^Department of Endocrinology, First Hospital of Qinhuangdao, Qinhuangdao, China; ^3^Health Management Centre, First Hospital of Qinhuangdao, Qinhuangdao, China; ^4^Department of Traditional Chinese Medicine, First Hospital of Qinhuangdao, Qinhuangdao, China; ^5^Department of Cardiology, First Hospital of Qinhuangdao, Qinhuangdao, China

**Keywords:** depressive symptoms, sugar intake, obesity, cross-sectional study, NHANES

## Abstract

**Background:**

The existing literature has repeatedly assessed the association between sugar-sweetened beverages and depressive symptoms, but studies of the association of total dietary sugar with depressive symptoms and of this association in obese adults are scarce. Thus, the purpose of this cross-sectional study was to assess the association between total sugar consumption and depressive symptoms in the study population and then in the population stratified by body mass index.

**Methods:**

This study was conducted in a nationally representative sample of 16,009 adults from the 2011–2018 National Health and Nutrition Examination Survey in the US. Total sugar intake was assessed by 24 h dietary recalls, and depressive symptoms were assessed by the nine-item Patient Health Questionnaire. Logistic regression models were used to evaluate the association between total sugar consumption and depressive symptoms.

**Results:**

Total sugar intake was positively associated with higher prevalence of depressive symptoms, and the adjusted odds ratio (95% confidence interval) of depressive symptoms for the highest vs. lowest quintile of total sugar intake was 1.56 (1.18, 2.05). In stratified analysis, we found a positive association between total sugar intake and depressive symptoms in adults with body mass index ≥30 kg/m^2^ (*P* for trend = 0.013), whereas no association was found in normal weight or overweight adults.

**Conclusions:**

A higher intake of total sugar was associated with increased odds of clinically relevant depressive symptoms among obese adults. Further studies are necessary to confirm the role of total sugar in depressive symptoms among obese adults.

## 1. Introduction

Depression is a primary cause of disability and a significant contributor to the global burden of disease (1). According to the World Health Organization, depression affects more than 350 million people worldwide (2), and this disorder disproportionately affects individuals with obesity. The prevalence of depressive symptoms in obese people is twice as high as that in individuals of normal weight (3). According to the Global Burden of Disease Study 2019, obesity is also an important contributor to the global burden disease (4). Obesity as a major risk factor for non-communicable diseases is associated with reduced quality of life and life expectancy (5). The underlying cause of obesity is an energy imbalance between calories consumed and calories expended (5). There is evidence that diets high in sugar promote the development of obesity (6). Sugar consumption is strongly associated with the rise of obesity. In addition, obesity and depressive symptoms have a bidirectional relationship (2, 3, 7), as risk factors for depressive symptoms are also linked to the development of obesity (7). A meta-analysis concluded that obese individuals had a 55% increased risk of developing depressive symptoms, while depressed individuals had a 58% increased risk of becoming obese (7).

Although the pathogenesis of depressive symptoms is not well understood, there is evidence that nutrition and diet play important roles in the development of depressive symptoms. In recent years, a growing number of studies have explored the relationship between sugary foods/beverages and depressive symptoms (8–11). However, most studies focused on sugar-sweetened beverages rather than dietary total sugar intake, and the results were inconsistent. A meta-analysis of 10 observational studies indicated that consumption of sugar-sweetened beverages was associated with a higher prevalence of depressive symptoms (8). In contrast, the Seguimiento Universidad de Navarra project, which was a 10-year follow-up study involving 15,546 participants, found no significant association between the consumption of sugar-sweetened beverages and depressive symptoms (9). The association between total sugar intake and depressive symptoms has rarely been studied, with only two reports on this topic available to date. The Whitehall II cohort study found that high sugar intake was positively associated with depressive symptoms (10). However, the analysis was conducted in a sample of non-industrial civil servants aged 39–83 years, which limits the generalization of their findings. A similar positive association was found in a cross-national study involving six countries (11), but it did not take into account any confounding factors such as gender, physical activity, marital status, education level, family income, body mass index (BMI), etc. There is evidence that depressive disorders are twice as common in women as in men (12). Sociocultural roles, vulnerability to life events, and coping skills are reasons why women are more likely to suffer from depression. Besides, the evidence suggests that physical activity has a beneficial effect on depressive symptoms (13). Living with a spouse, higher levels of education and higher economic income are also associated with a lower prevalence of depressive symptoms too (12, 14, 15). Moreover, none of these studies focused on obese adults or conducted stratified analysis by BMI.

Therefore, the goal of the current study was to investigate the association between total sugar consumption and depressive symptoms in a cross-sectional study of US adults, adjusting for numerous confounding factors (including gender, physical activity, marital status, education level, family income, etc.). The data were further stratified by BMI to assess the patterns in obese adults.

## 2. Materials and methods

### 2.1. Study population

The data analyzed in this study came from the National Health and Nutrition Examination Survey (NHANES), which is a nationally representative survey conducted by the Centers for Disease Control and Prevention of the United States. The Research Ethics Review Board of the National Center for Health Statistics approved the NHANES study protocol, with all participants providing signed informed consent. From 2011 to 2018, a total of 39,156 individuals participated in the NHANES, and our analyses were confined to 23,826 individuals over the age of 18 years. Pregnant females (*n* = 247) and those who did not complete the depression scale (*n* = 3,289) were omitted from the study. Moreover, individuals with incomplete 24 h dietary recall data (*n* = 3,355) or with implausible energy intake (<500 or >5,000 kcal/day; *n* = 926) (16, 17) were further excluded. Finally, 16,009 participants were included in the analyses.

### 2.2. Assessment of depressive symptoms

The nine-item Patient Health Questionnaire (PHQ-9), a valid criteria instrument based on DSM-V, was used to measure depressive symptoms (18, 19). The PHQ-9 consists of nine items. The PHQ score of each participant is the sum of all answers to the PHQ question. Because a PHQ-9 score ≥10 points has 88% sensitivity and 88% specificity in diagnosing significant depression symptoms (18), it was chosen as the binary threshold to determine the existence of depressive symptoms in this study.

### 2.3. Assessment of dietary intake

Two 24 h dietary recall interviews were used to examine dietary data. The first interview was conducted in-person at the Mobile Examination Center, and the second interview was conducted over the phone 3–10 days later. The interviewer inquired about all foods and beverages ingested in the previous 24 h. To assist respondents in reporting food amounts during the interview, participants were given a set of measurement instructions and a food model booklet. The Food and Nutrient Database for Dietary Studies was used to calculate dietary intakes. Data from the two 24 h recalls were utilized to calculate the mean of dietary intake.

### 2.4. Covariates

The following covariates were included in this study: age, gender, BMI, energy intake, physical activity, marital status, race, education level, family income, smoking/drinking history, hypertension, and diabetes. Physical activity was assessed using a physical activity questionnaire based on the Global Physical Activity Questionnaire, which provided respondent-level interview data on physical activity. Total physical activity was calculated by weighting the metabolic equivalent of task (MET) minutes of each activity and adding them together. The MET score for each activity was suggested by the NHANES database. Marital status was categorized into three groups: married/living with partner, widowed/divorced/separated/never-married, and other. Race was categorized as Mexican American, other Hispanic, non-Hispanic White, non-Hispanic Black, and other. Education was divided into three levels: pre-high school, high school, and post-high school. Family income was categorized as <$20,000, $20,000–$74,999, and ≥$75,000. Alcohol status was evaluated according to the survey question “Had at least 12 alcohol drinks a year?”. Lifetime smoking of at least 100 cigarettes was considered as having a smoking history. Hypertension or diabetes was determined by a doctor's diagnosis.

### 2.5. Statistical analyses

Categorical variables were expressed by proportions (%) while continuous variables were expressed by the mean ± standard deviation or median (interquartile range). One-way analysis of variance, the Kruskal-Wallis *H*-test, or the chi-square test was used to analyze the distribution among the quintiles of dietary sugar intake according to the characteristics of the variables. Odds ratio (OR) and 95% confidence interval (CI) were calculated to assess the association between total sugar intake and depressive symptoms using logistic regression models. Model 1 was adjusted for age, gender, and BMI. Model 2 was fully adjusted, including age, gender, BMI, energy intake, physical activity, marital status, race, education level, family income, smoking/drinking history, hypertension, and diabetes. Restricted cubic spline (RCS) models were applied to assess the non-linear association between total sugar intake and depressive symptoms after adjusting for variables in Model 2. We further stratified the analyses by BMI (<25, 25–30, or ≥30 kg/m^2^) and gender (male or female). All analyses conducted in this study were weighted. All statistical analyses were carried out using R (http://www.R-project.org, The R Foundation), Free Statistics software, and EmpowerStats (20). A two-sided *P* < 0.05 was considered to be statistically significant.

## 3. Results

Among the 16,009 participants, 7,723 males and 8,286 females were eligible for the final analyses, and 1,365 (8.53%) reported depressive symptoms. Table 1 summarizes the characteristics of the study participants by categories of total sugar intake. Adults with higher intake of sugar tended to be younger and male, and they had higher levels of energy intake and physical activity. Statistically significant differences were also observed for marital status, race, educational level, family income, smoking and drinking status, and history of diabetes and hypertension (all *P* < 0.05). No significant difference was observed for BMI (*P* > 0.05).

**Table 1:** Participant characteristics according to sugar intake.

**Characteristics**	**Categories of total sugar intake(g/day)**					**P-value**
	**Level 1**	**Level 2**	**Level 3**	**Level 4**	**Level 5**	
No. of participants	3,202	3,202	3,200	3,203	3,202	–
Depressive symptoms (%)	8.32	6.11	6.73	7.92	9.21	<0.001
Age (years)	49.02 ± 17.10	48.55 ± 17.83	49.14 ± 17.78	47.22 ± 17.60	45.71 ± 16.80	<0.001
Gender (male, %)	39.39	41.69	44.38	51.79	62.24	<0.001
BMI (kg/m^2^)	29.67 ± 6.99	29.21 ± 6.91	29.39 ± 6.96	29.12 ± 6.97	29.35 ± 7.37	0.351
Total energy intake (kcal/day)	1,505.93 ± 506.36	1,784.07 ± 536.18	1,997.32 ± 531.22	2,252.76 ± 579.35	2,710.69 ± 653.21	<0.001
Physical activity (MET-minutes/week)	1,280 (232, 3,680)	1,380 (200, 3,840)	1,320 (180, 3,440)	1,680 (280, 5,040)	1,920 (360, 6,660)	<0.001
**Marital status (%)**						
Married/living with partner	63.11	62.1	63.59	61.83	60.37	0.019
Widowed/divorced/separated/Never-married	34.57	34.87	33.3	34.66	35.92	
Other	2.33	3.03	3.1	3.51	3.71	
**Race (%)**						
Mexican American	7	7.81	7.97	9.24	7.9	<0.001
Other Hispanic	5.78	5.15	6.52	5.89	5.62	
Non-Hispanic White	67.54	67.57	67	66.54	69.55	
Non-Hispanic Black	9.52	10.32	10.87	11.29	11.32	
Other race	10.17	9.15	7.64	7.04	5.62	
**Education level (%)**						
Below high school	4.29	3.46	3.12	3.3	3.27	0.010
High school	93.4	93.53	93.8	93.18	93.01	
Above high school	2.31	3.01	3.09	3.51	3.71	
**Family income (%)**						
<$20,000	14.53	14.48	13.34	14.95	17.29	<0.001
$20,000–$74,999	48.66	48.84	48.01	51.18	52.29	
≥$75,000	36.81	36.69	38.65	33.87	30.42	
**Smoking status (%)**						
Yes	43.66	40.82	38.74	40.42	46.12	<0.001
No	56.34	59.18	61.26	59.58	53.88	
**Drinking status (%)**						
Yes	54.38	56.47	56.94	57.98	60.18	<0.001
No	14.62	17.83	17.59	17.57	16.52	
Not recorded	31	25.7	25.47	24.45	23.3	
**Hypertension (%)**						
Yes	37.42	32.56	33.13	30.35	30.67	<0.001
No	62.58	67.44	66.87	69.65	69.33	
**Diabetes (%)**						
Yes	14.58	12.71	10.24	8.34	6.96	<0.001
No	85.42	87.29	89.76	91.66	93.04	

BMI, body mass index; MET, metabolic equivalent of task.

**Figure 1:**
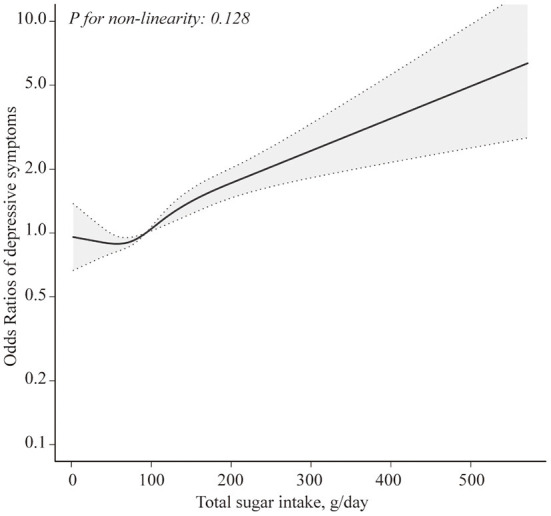
Restricted cubic spline model of the odds ratios of total sugar intake with depressive symptoms. Adjusted for age, gender, BMI, total energy intake, physical activity, marital status, race, education level, household income, smoking status, drinking history, hypertension, and diabetes. The dashed lines represent the 95% confidence intervals.

Table 2 presents the association between total sugar intake and depressive symptoms. In the crude model, total sugar intake was positively associated with depressive symptoms (*P* for trend = 0.047). After multivariable adjustments, the positive association remained significant (*P* for trend = 0.001). Compared with the participants in the lowest quintile, the ORs (95% CIs) across increasing intake of total sugar were 0.81 (0.61, 1.06), 1.00 (0.76, 1.32), 1.25 (0.94, 1.66), and 1.56 (1.18, 2.05). To assess the dose-response association between sugar intake and depressive symptoms, RCS analysis was conducted. Figure 1 shows a linear association between sugar intake and depressive symptoms in all participants (*P* for non-linearity = 0.128).

**Table 2:** Association between total sugar intake and depressive symptoms.

	**Categories of total sugar intake (g/day)**					**P for trend**
	**Level 1**	**Level 2**	**Level 3**	**Level 4**	**Level 5**	
No. of participants	3,202	3,202	3,200	3,203	3,202	–
No. of participants with depressive symptoms	277	229	244	284	331	–
Crude model	Ref	0.72 (0.55, 0.93)	0.80 (0.62, 1.02)	0.95 (0.75, 1.19)	1.12 (0.94, 1.33)	0.047
Adjusted model 1	Ref	0.73 (0.56, 0.95)	0.82 (0.63, 1.06)	1.02 (0.81, 1.30)	1.26 (1.05, 1.52)	0.004
Adjusted model 2	Ref	0.81 (0.61, 1.06)	1.00 (0.76, 1.32)	1.25 (0.94, 1.66)	1.56 (1.18, 2.05)	0.001

BMI, body mass index.

Multiple logistic regression analysis.

Adjusted model 1 adjusted for age, gender, and BMI.

Adjusted model 2 adjusted for age, gender, BMI, total energy intake, physical activity, marital status, race, education level, household income, smoking status, drinking history, hypertension, and diabetes.

Table 3 shows the result of stratified analysis by BMI. In obese adults (BMI > 30 kg/m^2^), after multivariable adjustments, total sugar intake was positively associated with depressive symptoms (*P* for trend = 0.013). The corresponding ORs (95% CIs) were 1 (reference), 0.87 (0.61, 1.25), 1.04 (0.70, 1.55), 1.27 (0.85, 1.89), and 1.66 (1.13, 2.46). However, among adults with BMI <30 g/m^2^, no significant association between total sugar intake and depressive symptoms was found in any of the models. When RCS analyses were conducted in normal weight, overweight, and obese adults, respectively, the results did not indicate evidence against linearity (all *P* for non-linearity > 0.05) (Supplementary Figure 1).

**Table 3:** Associations between total sugar intake and depressive symptoms stratified by BMI.

	**Categories of total sugar intake (g/day)**					**P for trend**

	**Level 1**	**Level 2**	**Level 3**	**Level 4**	**Level 5**	
**BMI**<**25 kg/m**^2^ **(*****n*** = **4,584)**						
No. of participants	917	917	916	917	917	–
No. of participants with depressive symptoms	57	53	59	60	82	–
Crude model	Ref	0.62 (0.37, 1.05)	0.85 (0.47, 1.51)	0.76 (0.49,1.19)	1.11 (0.81, 1.54)	0.263
Adjusted model 1	Ref	0.62 (0.37, 1.05)	0.84 (0.47, 1.52)	0.76 (0.48, 1.19)	1.09 (0.76, 1.57)	0.355
Adjusted model 2	Ref	0.77 (0.44, 1.34)	1.07 (0.55, 2.08)	1.12 (0.68, 1.85)	1.58 (0.86, 2.89)	0.065
**25** ≤ **BMI**<**30 kg/m**^2^ **(*****n*** = **5,072)**						
No. of participants	1,015	1,013	1,015	1,014	1,015	–
No. of participants with depressive symptoms	71	50	51	68	79	–
Crude model	Ref	0.59 (0.36, 0.99)	0.64 (0.40, 1.03)	0.97 (0.64, 1.45)	1.14 (0.78, 1.66)	0.162
Adjusted model 1	Ref	0.59 (0.35, 1.00)	0.66 (0.42, 1.05)	1.03 (0.69, 1.53)	1.27 (0.87, 1.86)	0.069
Adjusted model 2	Ref	0.65 (0.38, 1.11)	0.76 (0.47, 1.23)	1.13 (0.74, 1.73)	1.25 (0.70, 2.22)	0.193
**BMI** ≥**30 kg/m**^2^ **(*****n*** = **6,353)**						
No. of participants	1,271	1,270	1,271	1,270	1,271	–
No. of participants with depressive symptoms	148	130	131	158	168	–
Crude model	Ref	0.86 (0.62, 1.20)	0.82 (0.58, 1.16)	1.02 (0.74, 1.42)	1.13 (0.87, 1.48)	0.240
Adjusted model 1	Ref	0.86 (0.62, 1.19)	0.85 (0.59, 1.20)	1.10 (0.79, 1.53)	1.32 (1.01, 1.74)	0.037
Adjusted model 2	Ref	0.87 (0.61, 1.25)	1.04 (0.70, 1.55)	1.27 (0.85, 1.89)	1.66 (1.13, 2.46)	0.013

BMI, body mass index.

Multiple logistic regression analysis.

Adjusted model 1 adjusted for age, and gender.

Adjusted model 2 adjusted for age, gender, total energy intake, physical activity, marital status, race, education level, household income, smoking status, drinking history, hypertension, and diabetes.

Table 4 shows the associations between total sugar intake and depressive symptoms by gender in normal weight, overweight, and obese adults. In obese adults (BMI ≥30 kg/m^2^), after full adjustments, total sugar intake was positively associated with depressive symptoms in both females (*P* for trend = 0.034) and males (*P* for trend = 0.015). For those with BMI <30 g/m^2^, there was no association between total sugar consumption and depressive symptoms in either females or males.

**Table 4:** Associations between total sugar intake and depressive symptoms by gender in normal weight, overweight and obese adults.

	**Categories of total sugar intake (g/day)**					**P for trend**

	**Level 1**	**Level 2**	**Level 3**	**Level 4**	**Level 5**	
**BMI**<**25 kg/m**^2^						
**Females (*****n*** = **2,469)**						
No. of participants	494	494	493	494	494	–
No. of participants with depressive symptoms	30	29	32	43	49	–
Crude model	Ref	0.85 (0.42, 1.71)	0.99 (0.45, 2.21)	1.14 (0.64, 2.03)	1.25 (0.72, 2.17)	0.229
Adjusted model	Ref	1.06 (0.50, 2.21)	1.24 (0.53, 2.89)	1.49 (0.78, 2.80)	1.70 (0.90, 3.20)	0.056
**Males (*****n*** = **2,115)**						
No. of participants	422	424	423	423	423	–
No. of participants with depressive symptoms	30	17	23	25	33	–
Crude model	Ref	0.25 (0.09, 0.67)	0.67 (0.32, 1.43)	0.49 (0.28, 0.86)	0.63 (0.36, 1.09)	0.485
Adjusted model	Ref	0.28 (0.09, 0.88)	0.90 (0.41, 1.99)	0.65 (0.31, 1.36)	0.93 (0.36, 2.38)	0.724
**25** ≤ **BMI**<**30 kg/m**^2^						
**Females (*****n*** = **2,230)**						
No. of participants	446	446	446	446	446	–
No. of participants with depressive symptoms	34	34	30	38	43	–
Crude model	Ref	0.68 (0.35, 1.32)	0.74 (0.39, 1.37)	0.98 (0.56, 1.71)	1.27 (0.75, 2.15)	0.246
Adjusted model	Ref	0.72 (0.36, 1.40)	0.81 (0.43, 1.51)	1.20 (0.64, 2.25)	1.19 (0.50, 2.83)	0.462
**Males (*****n*** = **2,842)**						
No. of participants	569	568	568	568	569	–
No. of participants with depressive symptoms	31	21	17	26	45	–
Crude model	Ref	0.61 (0.28, 1.31)	0.59 (2.67, 1.31)	1.03 (0.48, 2.19)	1.26 (0.67, 2.36)	0.231
Adjusted model	Ref	0.66 (0.31, 1.40)	0.66 (0.30, 1.46)	1.11 (0.54, 2.32)	1.03 (0.44, 2.44)	0.570
**BMI** ≥**30 kg/m**^2^						
**Females (*****n*** = **3,587)**						
No. of participants	718	717	717	717	718	–
No. of participants with depressive symptoms	104	80	98	103	129	–
Crude model	Ref	0.78 (0.51, 1.20)	0.95 (0.62, 1.47)	0.95 (0.64, 1.41)	1.33 (0.93, 1.91)	0.099
Adjusted model	Ref	0.83 (0.54, 1.27)	1.20 (0.75, 1.90)	1.14 (0.70, 1.86)	1.76 (1.10, 2.82)	0.034
**Males (*****n*** = **2,766)**						
No. of participants	553	553	553	553	554	–
No. of participants with depressive symptoms	42	40	32	46	61	–
Crude model	Ref	0.80 (0.41, 1.54)	0.72 (0.39, 1.34)	1.18 (0.60, 2.30)	1.67 (0.80,3.49)	0.112
Adjusted model	Ref	0.80 (0.39, 1.63)	0.89 (0.44, 1.81)	1.59 (0.84, 3.04)	2.37 (1.09, 5.17)	0.015

BMI, body mass index.

Multiple logistic regression analysis.

Adjusted model adjusted for age, total energy intake, physical activity, marital status, race, education level, household income, smoking status, drinking history, hypertension, and diabetes.

## 4. Discussion

This nationally representative cross-sectional study demonstrated a positive association between total sugar intake and depressive symptoms in adults when adjusted for potentially important confounders. A similar significant association was observed in obese females and males, but not in normal weight or overweight adults. To the best of our knowledge, this is the first study to explore the association between total sugar intake and depressive symptoms in a stratified analysis based on BMI in the US adult population.

In previous studies, researchers evaluated the association between sugary foods/beverages and depressive symptoms (8, 21, 22). A meta-analysis of 10 observational studies including 365,289 participants indicated that drinking sugar-sweetened beverages was linked to a higher frequency of depression symptoms (8). Another two cross-sectional studies conducted in Brazilian adults and Chinese adolescents consistently showed a positive association between the consumption of sugar-sweetened beverage and depressive symptoms (21, 22). In other cases, the results were based on analyses of dietary patterns. The Whitehall II prospective cohort indicated that a diet rich in processed foods, such as chocolate, sweet desserts, processed meat, and fried food, was deleterious for depressive symptoms (OR = 1.58; 95% CI 1.11, 2.23) (23). In a case-control study, Khosravi et al. (24) showed that adherence to unhealthy dietary patterns, such as those defined by a high intake of sweets and commercial fruit drinks, increased the OR of depressive symptoms. In contrast to numerous studies of the effects of sugary foods/beverages on depressive symptoms, there are only two reports about the association between total sugar intake and depressive symptoms. The results of the Whitehall II study provided evidence that total sugar intake increased the risk of depressive symptoms in males (10). In addition, a study based on data from six countries reported a relationship between total sugar intake and the prevalence of major depression (11).

Although little is known about the underlying mechanisms of the positive association between sugar intake and depressive symptoms, various possibilities have been proposed. The pathophysiology of depressive symptoms includes dysregulation of the hypothalamic-pituitary-adrenal (HPA) axis, resulting in reduced volumes in the hippocampus, prefrontal cortex, and striatum (25), and the activity of the HPA axis can be inhibited *via* the consumption of dietary sugar (26). A recent review showed that the neuroadaptations that occurred in the hippocampus, prefrontal cortex, and amygdala after consuming sugar increased the likelihood of developing depressive symptoms (27). Inflammation is another potential biological explanation for the link between sugar consumption and depressive symptoms. In both humans (28) and rats (29), dietary sugar has been shown to induce proinflammatory states that are characterized by increased expression of inflammatory genes and raised levels of inflammatory factors. Inflammation is recognized as a potent physiological trigger of depressive symptoms, especially fatigue, lack of energy, sleep problems, and changes in appetite (30, 31). Anti-inflammatory drugs have antidepressant therapeutic characteristics, while pro-inflammatory drugs may induce depressive symptoms and raise the likelihood of developing full-blown depressive symptoms (32). Excessive sugar consumption can also drive insulin resistance and impair glucose homoeostasis. Previous studies demonstrated that insulin resistance increased the risk for the development of future depressive symptoms, and higher insulin resistance biomarkers were more prevalent in those who reported having depressive symptoms (33, 34). Evidence also revealed that insulin resistance in the brain and the consequent disruption in energy use might be a direct cause of depressive symptoms (32).

Given that the relationships among sugar intake, depressive symptoms, and obesity are of particular interest, data were further stratified by BMI. Total sugar intake was shown to be associated with higher prevalence of depressive symptoms in obese adults. Several previous studies suggested a bidirectional association between depressive symptoms and obesity (2, 7, 35). A meta-analysis of eight longitudinal studies concluded that obese individuals had a 55% increased risk of developing depressive symptoms, whereas depressed people had a 58% increased risk of becoming obese (7). Furthermore, studies of both humans and animals have demonstrated a connection between depressive symptoms and sweet desires (36). Sweet foods can enhance mood and relieve negative emotional states (27), but excessive sugar intake is one of the leading contributors to weight gain (37), and obesity has been found to increase risk of depressive symptoms. These results suggest a tight link among dietary sugar, depressive symptoms, and obesity. The association between sugar consumption and depressive symptoms may be mediated by obesity, as obesity can activate inflammatory pathways, increase risk of insulin resistance, and contribute to HPA-axis dysregulation (7, 38, 39), all of which may be involved in depressive symptoms.

The co-existence of depressive symptoms with obesity has raised as an important public health issue. When obese adults with depressive symptoms consult a dietitian/doctor for weight loss, it is crucial that they receive scientific dietary advice. Losing weight and easing depressed mood at the same time by adjusting diet is a win-win approach. However, research in this field is scarce. Studies of the association between sugar consumption and depression symptoms have mostly focused on sugar-sweetened drinks, with little attention paid to total sugar intake, and studies of the association between total sugar intake and depressive symptoms in obese adults are extremely rare. The findings of our study can be utilized to provide a scientific basis for gradually reducing sugar intake in obese adults with depressive symptoms. For these adults, good diet and lifestyle habits can help control weight while alleviating depressed mood. We should do more to reduce total sugar consumption through public policy. Besides energy intake control, energy expenditure constitutes the other important factor in the energy balance formula. Physical activity can help with energy expenditure. However, strong evidence reveals that insufficient physical activity presents a major global public health issue. In 2019, low physical activity potentially contributed to 0.83 million deaths and 15.75 million disability-adjusted life years globally (40). Therefore, the dissemination of appropriate diet and physical activity recommendations is crucial today.

The current study has several strengths. First, we used a large nationally representative sample, which provided sufficient statistical power. We also included and adjusted for numerous confounding factors, including sociodemographic, behavioral, anthropometric, and clinical factors. To the best of our knowledge, this study is the first to investigate the association between total sugar consumption and depressive symptoms in a stratified analysis based on BMI. Possible limitations of this study are as follows: (1) due to the cross-sectional design, causal association between dietary sugar and depressive symptoms cannot be confirmed; (2) although the study used two 24 h dietary recalls to assess total food intake in a detailed way, recall bias and day-to-day variation are unavoidable; (3) the PHQ-9 scores do not correspond with a clinical diagnosis of depression but rather indicate the level of depressive symptoms that may be of clinical relevance; and (4) even though a variety of confounders were considered, we cannot rule out the possibility that some unknown or unmeasured factors might partly explain the results. For example, the NHANES database did not assign METs by age groups, which might lead to bias.

## 5. Conclusions

In summary, we identified a positive association between total sugar intake and prevalence of depressive symptoms in obese adults. Further cohort studies and randomized controlled trials are needed to provide more powerful evidence to elucidate the association.

## Data availability statement

The original contributions presented in the study are included in the article/Supplementary material, further inquiries can be directed to the corresponding author.

## Ethics statement

The studies involving human participants were reviewed and approved by Research Ethics Review Board of the National Center for Health Statistics. The patients/participants provided their written informed consent to participate in this study.

## Author contributions

The study was conceived and designed by PL and JZ. The data was examined by PL, YZ, RZ, and QW. The manuscript was drafted and revised by PL, FY, YL, JW, WL, and QW. FY, QW, and JZ supervised the analyses and suggested revisions of the paper. The final paper has been approved by all the authors.
