# Supplementary figures and images for: Total sugar intake is associated with higher prevalence of depressive symptoms in obese adults
Source: Front Public Health. 2023 Jan 13;10:1069162. doi: 10.3389/fpubh.2022.1069162 (PMC9880186; doi:10.3389/fpubh.2022.1069162)

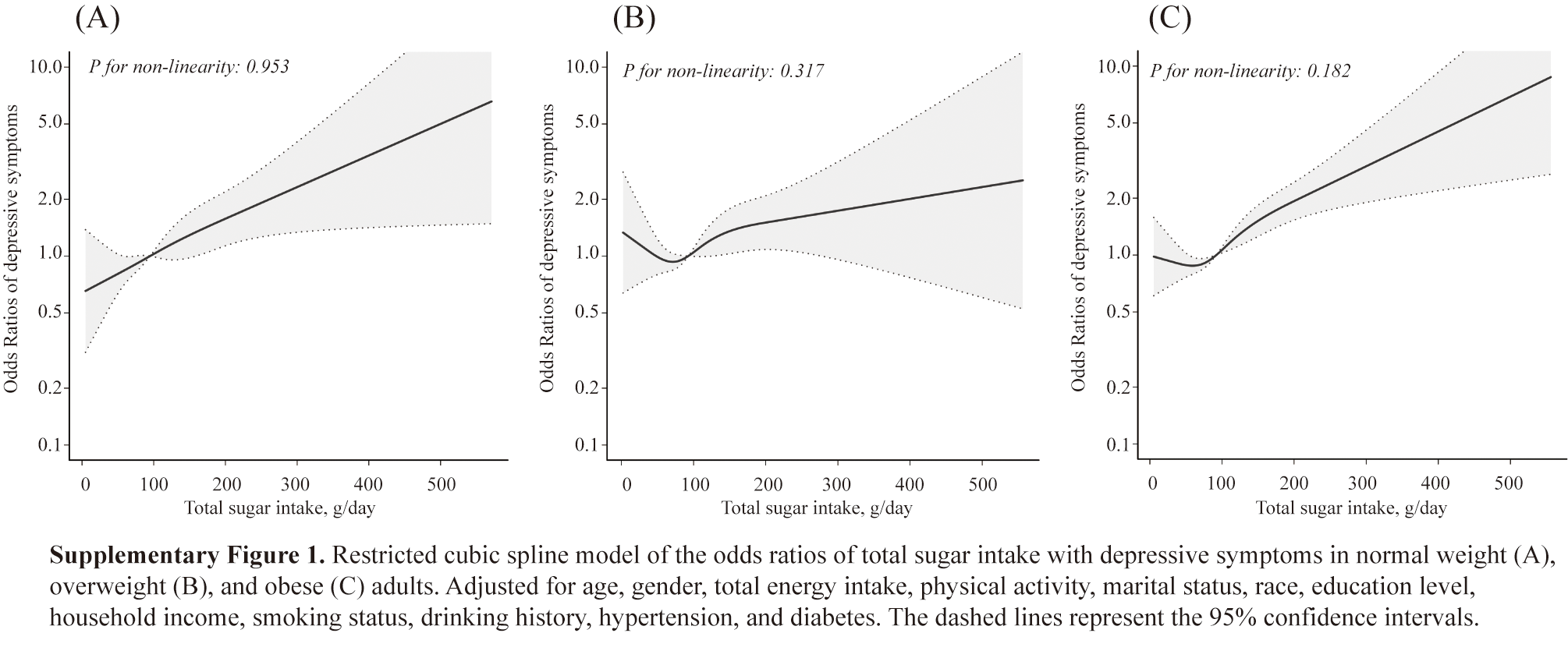

Supplement: Supplementary file 1 [file Image_1.TIF]
